# Steatosis worsens ischemia-reperfusion injury post hepatectomy with intermittent inflow occlusion

**DOI:** 10.1016/j.clinsp.2026.101151

**Published:** 2026-09-19

**Authors:** Hui Chen, Biao Duan, Shiyu Yang, Nuo Pan, Kun Tao, Xiaohui Liu, Bing Hu, Jun Chen, Weifeng Tan

**Affiliations:** a Department of Endoscopy, Eastern Hepatobiliary Hospital, Second Military Medical University, Shanghai, China; b Department of Hepatobiliary and Pancreatic Surgery, Tongji Hospital Affiliated to Tongji University, Shanghai, 200333, China; c Baoshan Branch of Renji Hospital, Shanghai Jiao Tong University School of Medicine, Shanghai, China; d Kangda College, Nanjing Medical University, Jiangsu province, Lianyungang, China; e Department of Pathology, Tongji Hospital Affiliated to Tongji University, Shanghai, China

**Keywords:** Hepatectomy, Vascular inflow occlusion, Liver ischemia-reperfusion injury, Steatosis, Hepatoprotective

## Abstract

**Purpose:**

Intermittent Vascular Inflow Occlusion (VIO) is widely used during hepatectomy, but its impact on Liver Ischemia-Reperfusion Injury (LIRI) – especially in steatotic livers – remains debated. This study aims to evaluate the effects of VIO and hepatic steatosis on LIRI severity and postoperative recovery after hepatectomy.

**Methods:**

A retrospective cohort study analyzed 200 patients undergoing hepatectomy. Patients were divided into three groups according to the number of hepatic inflow occlusion episodes: N-VIO (no occlusion, n = 136), F-VIO (few-VIO, 1–2 occlusions, n = 35), and M-VIO (multiple-VIO, ≥ 3 occlusions, n = 29). After 1:1:1 Propensity Score Matching (PSM) based on surgical approach and tumor characteristics, 23 matched triplets were generated among the three groups. Liver function (ALT, AST) was assessed preoperatively and on Postoperative Days-3 (POD3) and 7 (POD7). Steatosis effects were evaluated.

**Results:**

At POD3, the three groups exhibited a trending overall difference in ALT levels before PSM (p = 0.009). Following PSM, no significant intergroup differences were observed at either POD3 (p = 0.422) or POD7 (p = 0.156). Collectively, these results suggest that, within the confines of the present study, variations in the number of hepatic inflow occlusions do not translate into significant differences in early postoperative ALT recovery. Steatosis significantly exacerbated IRI, with higher ALT in steatosis patients at POD3 (p = 0.026) and POD7 (p = 0.006).

**Conclusions:**

Appropriate VIO does not exacerbate LIRI. Steatosis heightens LIRI susceptibility. Patients with liver steatosis should pay more attention to controlling the number of VIO during the operation.

## Highlights

• Vascular Inflow Occlusion (VIO) doesn’t exacerbate post-hepatectomy liver injury.
• Hepatic steatosis increases susceptibility to ischemia-reperfusion injury.
• VIO safe in non-steatotic livers, but caution needed for steatotic patients.
• Steatotic livers: stricter limits on occlusion cycles and duration.

## Introduction

Hepatectomy, is a commonly applied surgical technique to remove segments, lobes, or parts of the liver in cases of localized hepatic lesions. These lesions may include hepatic tumors, intrahepatic bile duct stones, hepatic trauma, and abscesses.^1^ Hepatectomy is widely used due to its effectiveness in treating a variety of liver conditions, especially hepatic malignancies.^2^^,^^3^

Despite the wide use of hepatectomy, the liver’s complex vascular anatomy and rich blood supply pose a major challenge for surgeons, particularly with regards to controlling intraoperative bleeding. To address this issue, various vascular occlusion techniques have been developed, with the primary aim of minimizing blood loss during surgery.^4^ The Pringle Maneuver (PM) involves clamping the hepatoduodenal ligament, thereby temporarily obstructing the liver's blood inflow.^5^, ^6^, ^7^ In Intermittent Pringle Maneuver (IPM) technique, blood inflow is occluded for 15-minute intervals, followed by 5-minute reperfusion periods to allow the liver to recover from ischemia.^8^^,^^9^ Although the IPM has shown efficacy in controlling blood loss, there is ongoing debate regarding its optimal use in terms of occlusion time and frequency, as well as its effectiveness in preventing Liver Ischemia-Reperfusion Injury (LIRI) compared to continuous occlusion.^10^, ^11^, ^12^ Indeed, while intermittent occlusion has been shown to extend the liver’s ischemic tolerance, the long-term effects of repeated ischemia-reperfusion cycles on liver recovery and overall prognosis are not fully understood.

Previous study found that patients with chronic liver disease were at a higher risk of liver failure when subjected to vascular occlusion, highlighting the vulnerability of diseased livers to ischemic insult.^13^ Also, livers with pre-existing conditions such as cirrhosis are more susceptible to damage after 60-minutes of occlusion.^14^^,^^15^ Intermittent occlusion strategies have been shown to reduce the severity of Ischemia-Reperfusion Injury (IRI), thus extending the liver’s ischemic tolerance.^16^ Some studies have reported that the maximum duration of hepatic Vascular Inflow Occlusion (VIO) can reach up to 350-minutes using IPM without significant increases in postoperative complications.^17^^,^^18^ However, the sensitivity of chronic liver disease to ischemic injury remains a major limitation, as these patients are less tolerant of both intermittent and continuous occlusion compared to those with healthy livers.^19^ As living conditions improve, the proportion of livers developing steatosis is increasing, and in addition to cirrhosis, steatosis is likely to be an important factor affecting the liver's tolerance to LIRI after hepatectomy.^20^ For these reasons, it is essential to explore the balance between occlusion duration and times to minimize the risk of liver failure and improve postoperative recovery in patients with chronic liver disease.

Given the complexities surrounding the use of VIO in hepatic surgery, this study was designed to investigate the effects of VIO on postoperative outcomes. We specifically aimed to explore the influence of steatosis on these outcomes. This study provides valuable insights into the tolerance of steatosis patient to ischemia and the potential for optimizing surgical techniques to improve postoperative recovery.

## Methods

### *Study design and participants*

This retrospective cohort study at the Hepatobiliary Surgery Center of Shanghai Tongji Hospital examined the impact of intraoperative VIO on postoperative liver function and prognosis in hepatic resection patients. Between September 6, 2022, and December 15, 2025, 202 patients were screened, with 200 meeting the inclusion criteria (aged 18‒80, scheduled for elective hepatic resection). Two were excluded due to age. Patients were divided into three groups according to the number of hepatic inflow occlusion episodes: N-VIO (no occlusion, n = 136), F-VIO (1–2 occlusions, n = 35), and M-VIO (≥ 3 occlusions, n = 29). Propensity score matching (1:1:1 ratio) based on surgical approach and tumor characteristics yielded 23 matched triplets across the three groups, with 23 patients retained in each group. Patients were also stratified by liver disease status. The study was ethically approved (K-2024–055), with informed consent obtained from all participants.

### *Data collection*

Clinical and demographic data were collected from all patients included in the study. The data collection included preoperative, intraoperative, and postoperative variables. Preoperative data included age, sex, Body Mass Index (BMI), length of hospital stay, and any pre-existing liver disease. Preoperative liver function tests were also recorded, including Total Bilirubin (TBIL), Direct Bilirubin (DBIL), Total Protein (TP), Albumin (ALB), Alanine Aminotransferase (ALT), Aspartate Aminotransferase (AST), Gamma-Glutamyl Transferase (GGT), and Alkaline Phosphatase (ALP). Intraoperative data encompassed the type of hepatic resection, duration of surgery, intraoperative blood loss, and the number of hepatic inflow occlusion episodes. VIO was performed at the surgeon’s discretion during the procedure, depending on the intraoperative bleeding status and the difficulty of parenchymal transection, rather than as a preplanned strategy. This aoperative allowed analysis of the surgical complexity and duration in relation to postoperative outcomes. Postoperative data were collected at two intervals: Postoperative Days-3 (POD3) and 7 (POD7). The same liver function markers were measured to evaluate the effects of VIO on liver injury and recovery.

### *H&E staining*

Formalin-fixed, paraffin-embedded liver tissue samples from all 64 patients who underwent hepatic inflow occlusion were sectioned and routinely stained with Hematoxylin and Eosin (H&E). Histopathological assessment for hepatic steatosis was performed independently by two experienced pathologists. The presence of steatosis was defined by the finding of > 5% of hepatocytes containing macrovesicular fatty vacuoles.^21^

### *Statistical analysis*

All statistical analyses were performed using SPSS 26.0 software. PSM was performed to minimize selection bias using R software (version 4.5.2). Patients in the N-VIO, F-VIO, and M-VIO groups were matched at a 1:1:1 ratio with a caliper width of 0.02 based on key covariates including surgical approach and tumor characteristics, yielding 23 matched triplets (23 patients per group). The balance of covariates after matching was assessed and deemed satisfactory. Continuous variables were expressed as mean ± Standard Deviation (SD) or median with Interquartile Range (IQR), depending on the distribution of the data. Categorical variables were presented as frequencies and percentages. Normality of the data was assessed using the Shapiro-Wilk test. For unpaired comparisons among the three groups, continuous variables were analyzed using one-way ANOVA or the Kruskal-Wallis test, and categorical variables using the Chi-Square test or Fisher's exact test, as appropriate. When overall differences were significant, post-hoc pairwise comparisons were performed using independent Student's *t*-tests (for normally distributed continuous variables), Dunn's test (for non-normally distributed continuous variables), or Chi-Square tests (for categorical variables), all with Bonferroni correction. For the 1:1:1 PSM-generated matched triplets, overall paired comparisons among the three groups were performed using the Friedman test (continuous variables) or Cochran's *Q*-test (categorical variables). Subsequent pairwise paired comparisons were conducted using Wilcoxon signed-rank tests or McNemar's tests, also with Bonferroni adjustment. For paired analyses, paired *t*-tests or Wilcoxon signed-rank tests were used for continuous variables, and McNemar's test for categorical variables. All tests were two-sided, and p < 0.05 was considered statistically significant.

## Results

### *Study population*

A total of 202 hospitalized patients were screened for eligibility. After excluding 2 patients, 200 patients were enrolled in the study (Fig. 1). The primary goal of the study was to evaluate the effects of VIO during hepatectomy on postoperative liver function and recovery, particularly in relation to the presence of liver steatosis. Of the enrolled patients, 136 did not undergo VIO (N-VIO), 35 underwent 1–2 occlusions (F-VIO), and 29 underwent ≥ 3 occlusions (M-VIO). Based on surgical approach and tumor characteristics, 1:1:1 propensity score matching was performed among the three groups, generating 23 matched triplets. To analyze the impact of liver disease, these groups were also stratified based on liver disease status.

**Fig. 1:**
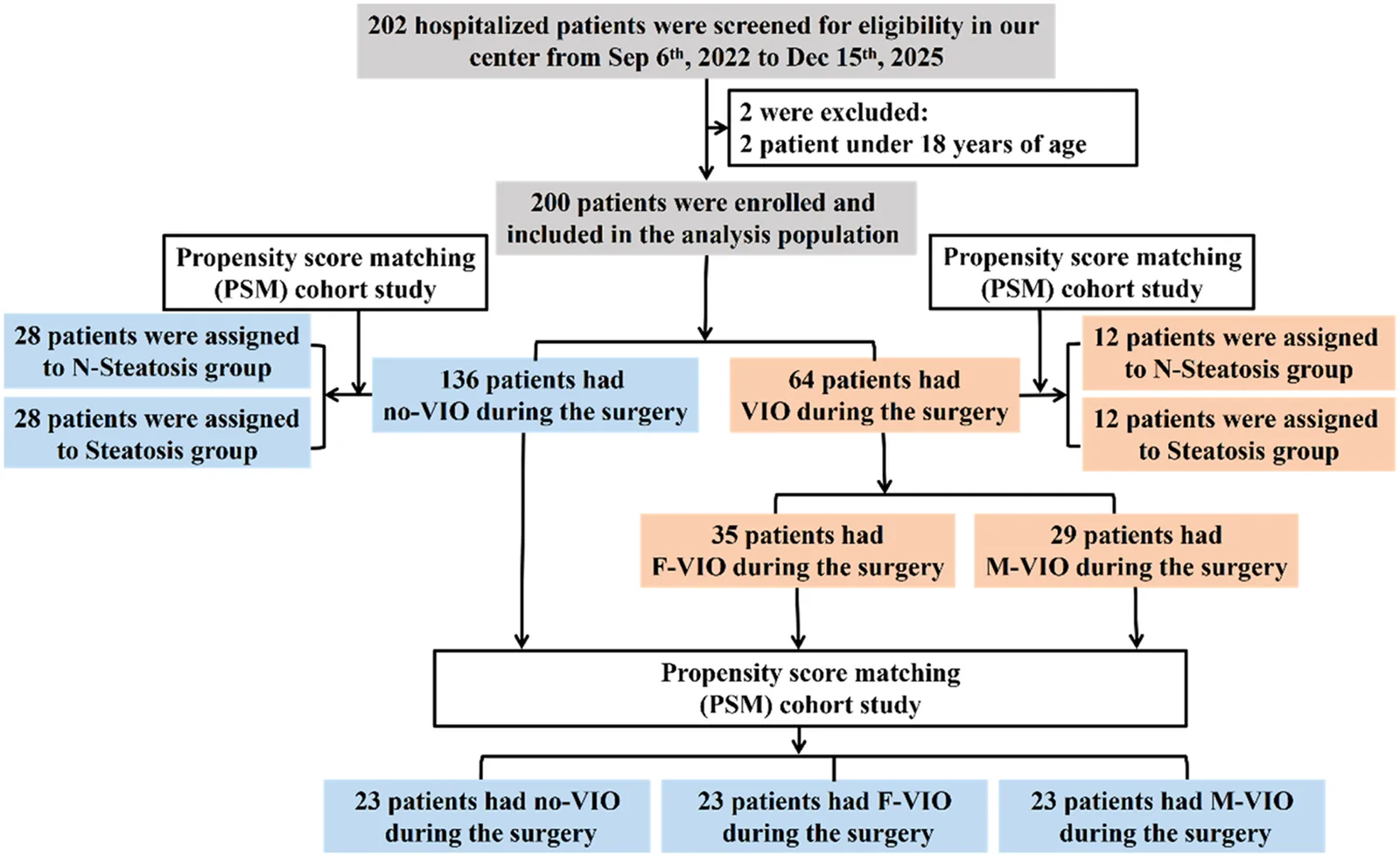
Diagram showing the flow of the patients in the cohort study.

Clinical characteristics, pre- and post- operative laboratory parameters were compared among the three groups (N-VIO, F-VIO, and M-VIO) of patients undergoing hepatectomy.

The clinical characteristics and preoperative laboratory values of patients undergoing hepatectomy were compared among the three groups (N-VIO, F-VIO, and M-VIO) (Table 1). Although the overall comparisons of TBIL and DBIL among the three groups reached statistical significance (p = 0.02 and p = 0.01, respectively), they are all within the normal reference range (TBIL, 5.1‒20.5 μmol/L and DBIL, < 6.8 μmol/L). Comparison of the level of ALT of patients showed that it was significantly elevated to different degrees, while they could basically return to the preoperative levels at POD7 (Table 1). Among them, especially in the M-VIO group, the elevation was more obvious at POD3. At POD3, the median ALT levels in the N-VIO, F-VIO, and M-VIO groups were 111.60 [47.85–216.00], 119.60 [44.65–230.60], and 215.60 [150.68–413.48] U/L, respectively. The overall difference among the three groups was statistically significant (p = 0.009). Post-hoc pairwise comparisons using Dunn's test with Bonferroni correction revealed that the M-VIO group had significantly higher ALT levels than the N-VIO group (p = 0.006), whereas no significant differences were observed between the N-VIO and F-VIO groups (p = 1.000) or between the F-VIO and M-VIO groups (p = 0.116). These findings suggest greater hepatocellular injury in the M-VIO group during the early postoperative period.

**Table 1:** Baseline characteristics of patients and liver function indexes of different postoperative phases in the following groups.

Characteristics	N-VIO group (n = 136)	F-VIO group (n = 35)	M-VIO group (n = 29)	p-value
Age (y)	62.5 [51‒69]	62 [56‒71]	60 [53.5‒72]	0.541
Sex (n)				0.012*
female	47.8% (65)	31.4% (11)	20.7% (6)	
male	52.2% (71)	68.6% (24)	79.3% (23)	
Amount of bleeding (mL)	150 [50‒400]	400 [200‒600]	400.00 [200‒725]	<0.001*
BMI (kg/m^2^)	22.83 [21.22‒24.53]	23.81 [20.86‒26.22]	25.35 [22.50‒28.08]	0.003*
Length of stay (d)	12 [7.25‒18]	12 [9‒17]	13 [9.5‒18]	0.612
Pre-existing liver disease				
Steatosis	20.6% (28)	17.1% (6)	20.7% (6)	0.897
Fibrosis/Cirrhosis	30.1% (41)	40.0% (14)	37.9% (11)	0.450
Liver tumor	52.9% (72)	71.4% (25)	89.7% (26)	<0.001*
Preoperative laboratory values				
TBIL (μmol/L)	15.10 [12.25‒22.10]	13.90 [10.20‒18.50]	13.30 [10.30‒16.75]	0.020*
DBIL (μmol/L)	3.40 [2.50‒5.65]	2.70 [1.90‒4.30]	2.70 [2.10‒3.78]	0.010*
TP (g/L)	67.60 [64.03‒71.98]	67.60 [63.50‒73.40]	68.60 [65.60‒71.70]	0.705
ALB (g/L)	38.89±4.50	39.20±4.14	40.11±3.37	0.378
ALT (U/L)	26.30 [16.53‒58.73]	23.90 [15.30‒46.90]	25.50 [19.50‒41.10]	0.803
AST (U/L)	28.90 [21.08‒48.28]	28.80 [19.60‒38.60]	30.70 [22.25‒41.95]	0.544
GGT (U/L)	54.00 [35.30‒151.50]	59.40 [24.00‒177.20]	52.00 [34.50‒101.73]	0.815
ALP (U/L)	106.00[79.60‒179.30]	104.50 [78.30‒154.50]	99.05 [67.45‒128.23]	0.176
POD3 laboratory values				
TBIL (μmol/L)	25.05 [17.25‒38.80]	15.10 [10.40‒26.25]	29.90 [15.48‒40.20]	0.005*
DBIL (μmol/L)	5.45 [3.18‒12.48]	3.15 [2.08‒7.48]	8.45 [2.40‒12.75]	0.068
TP (g/L)	62.05 [55,28‒67.03]	60.20 [56.75‒66.90]	56.05 [52.10‒62.78]	0.098
ALB (g/L)	34.50 [31.48‒37.25]	34.20 [31.35‒36.65]	33.45 [31.60‒37.05]	0.881
ALT (U/L)	111.60 [47.85‒216.00]	119.60 [44.65‒230.60]	215.60 [150.68‒413.48]	0.009*
AST (U/L)	98.45 [49.50‒175.03]	159.00 [36.55‒256.00]	186.00 [60.40‒359.75]	0.100
GGT (U/L)	52.25 [30.35‒113.20]	61.40 [28.50‒122.70]	59.90 [28.50‒115.15]	0.984
ALP (U/L)	97.50 [74.00‒158.80]	90.00 [71.00‒162.00]	82.40 [57.90‒125.20]	0.094
POD7 laboratory values				
TBIL (μmol/L)	16.80 [12.35‒29.33]	13.60 [12.00‒15.70]	15.50 [11.45‒26.83]	0.011*
DBIL (μmol/L)	3.60 [2.10‒9.95]	3.50 [2.05‒3.85]	4.00 [2.00‒7.00]	0.310
TP (g/L)	62.21±7.17	64.12±6.77	61.09±7.11	0.220
ALB (g/L)	34.10 [31.70‒36.30]	34.15 [31.53‒37.95]	35.00 [32.53‒36.38]	0.552
ALT (U/L)	50.00 [29.90‒87.50]	43.00 [26.50‒88.00]	83.90 [39.20‒126.33]	0.088
AST (U/L)	38.00 [24.18‒57.65]	37.85 [28.65‒41.25]	45.00 [27.00‒51.45]	0.372
GGT (U/L)	76.20 [50.00‒115.00]	73.80 [40.70‒128.90]	65.10 [45.10‒146.50]	0.856
ALP (U/L)	116.40 [84.10‒165.95]	116.40 [91.60‒125.70]	99.35 [74.88‒124.75]	0.174
Surgical procedure (n)				<0.001*
Hepatic cuneiform resection	46.3% (63)	22.9% (8)	0.0% (0)	
Segmentectomy	33.8% (46)	54.3% (19)	79.3% (23)	
Hemihepatectomy	19.9% (27)	22.9% (8)	20.7% (6)	
Duration of surgery (min)	211 [95‒358]	252 [207‒390]	280 [224.5‒319]	0.010*

Note: Data are presented as number (%) or means ± SD or median (1st quartile and 3rd quartile), as appropriate.

POD3, Postoperative Days-3; POD7, Postoperative Days-7; BMI, Body Mass Index; TBIL, Total Bilirubin; DBIL, Direct Bilirubin; TP, Total Protein; ALB, Albumin; ALT, Alanine Transaminase; AST, Aspartate Transaminase; GGT, Gamma-Glutamyl Transferase; ALP, Alkaline Phosphatase.

At POD7, the differences in ALT levels among the three groups (N-VIO, F-VIO, and M-VIO) were less pronounced, with no statistically significant pairwise differences. However, significant intergroup differences were observed in surgical approach and tumor characteristics, and the potential confounding effects of these factors on postoperative liver injury cannot be excluded.

To control for the significant baseline difference in surgical approach among the three groups, a PSM analysis was performed, yielding 23 well-matched triplets from the N-VIO, F-VIO, and M-VIO cohorts. In this balanced population, the differences in postoperative liver injury markers were attenuated and lost statistical significance (Table 2). At POD3, the median ALT levels in the N-VIO, F-VIO, and M-VIO groups were 125.00 U/L [58.00‒216.13], 138.50 U/L [36.60‒274.00], and 205.05 U/L [96.35‒396.70], respectively, with no statistically significant difference among the three groups (p = 0.422). Similarly, the median AST levels were 102.00 U/L [47.00‒201.00], 113.00 U/L [36.60‒281.90], and 180.50 U/L [42.88‒444.90], respectively, also showing no significant intergroup difference (p = 0.280). This finding suggests that the more pronounced liver injury observed in the initial analysis is more likely attributable to differences in the surgical procedure, with little evidence to support a substantial association between the number of VIO episodes and the extent of liver injury. Furthermore, VIO itself does not appear to significantly exacerbate postoperative liver function impairment. It highlights the critical importance of the surgeon's technical strategy in minimizing procedure-related hepatic injury during hepatectomy.

**Table 2:** Baseline characteristics of patients and liver function indexes of different postoperative phases in the following groups through PSM cohort study.

Characteristics	N-VIO group (n = 23)	F-VIO group (n = 23)	M-VIO group (n = 23)	p-value
Age (y)	59.74±12.19	60.91±11.35	61.04±12.71	0.757
Sex (n)				0.627
Female	34.8% (8)	26.1% (6)	21.7% (5)	
Male	65.2% (15)	73.9% (17)	78.3% (18)	
Intraoperative blood loss (mL)	200 [100‒500]	400 [300‒800]	400 [200‒800]	0.113
BMI (kg/m^2^)	22.63±2.01	23.62±3.41	25.31±3.17	0.007*
Length of stay (d)	21 [14‒25]	19 [16‒23]	19.00 [15‒28]	0.599
Pre-existing liver disease			13.00	
Steatosis	0.0% (0)	4.3% (1)	8.7% (2)	0.368
Fibrosis/Cirrhosis	21.7% (5)	34.8% (8)	21.7% (5)	0.500
Liver tumor	87.0% (20)	87.0% (20)	87.0% (20)	1.000
Preoperative laboratory values				
TBIL (μmol/L)	15.5 [11.1‒25.0]	13.1 [10.6‒18.5]	13.3 [9.9‒15.5]	0.296
DBIL (μmol/L)	3.50 [2.10‒6.80]	2.70 [2.00‒4.00]	2.40 [2.00‒3.33]	0.422
TP (g/L)	68.44±6.47	67.50±6.96	69.20±4.81	0.646
ALB (g/L)	38.29±4.09	38.88±3.48	39.99±3.20	0.278
ALT (U/L)	24.40 [14.80‒55.90]	23.30 [15.00‒43.60]	23.00 [19.10‒36.50]	1.000
AST (U/L)	26.90 [20.00‒42.73]	28.80 [20.00‒38.60]	30.70 [20.70‒42.80]	0.296
GGT (U/L)	68.30 [35.50‒134.20]	57.90 [28.40‒118.80]	56.55 [39.63‒105.60]	0.664
ALP (U/L)	106.40 [88.90‒179.30]	96.60 [78.30‒154.50]	94.15 [65.95‒120.50]	0.033*
POD3 laboratory values				
TBIL (μmol/L)	25.40 [12.20‒54.10]	16.50 [10.70‒28.40]	25.80 [11.33‒33.55]	0.610
DBIL (μmol/L)	5.20 [2.20‒15.20]	3.30 [1.95‒8.65]	7.80 [2.50‒13.50]	0.045*
TP (g/L)	62.40 [55.20‒67.30]	60.20 [56.70‒61.80]	55.70 [52.18‒66.95]	0.343
ALB (g/L)	34.01±3.40	34.02±4.61	35.08±4.48	0.620
ALT (U/L)	125.00 [58.00‒216.13]	138.50 [36.60‒274.00]	205.05 [96.35‒396.70]	0.422
AST (U/L)	102.00 [47.00‒201.00]	113.00 [36.60‒281.90]	180.50 [42.88‒444.90]	0.280
GGT (U/L)	66.30 [34.45‒109.40]	57.60 [24.20‒122.80]	64.60 [30.25‒116.90]	0.867
ALP (U/L)	92.10 [75.63‒161.80]	90.00[66.50‒153.90]	82.40 [58.20‒112.35]	0.368
POD7 laboratory values				
TBIL (μmol/L)	18.15 [12.98‒37.53]	14.00 [12.60‒15.90]	15.15 [11.40‒21.73]	0.089
DBIL (μmol/L)	3.60 [1.00‒7.05]	3.50 [2.20‒3.85]	4.10 [1.98‒5.75]	0.196
TP (g/L)	61.48±6.73	65.23±6.68	60.31±6.41	0.039*
ALB (g/L)	33.50 [30.18‒35.20]	35.80 [32.40‒39.30]	34.65 [32.78‒36.53]	0.021*
ALT (U/L)	70.00 [30.78‒141.00]	41.00 [24.00‒100.00]	83.90 [49.20‒120.78]	0.156
AST (U/L)	39.05 [22.78‒57.88]	37.20 [25.40‒42.00]	45.05 [29.40‒51.60]	0.953
GGT (U/L)	95.00 [75.45‒115.85]	58.60 [40.60‒111.50]	82.20 [43.20‒146.50]	0.472
ALP (U/L)	129.15 [100.33‒180.00]	112.40 [88.60‒134.60]	99.35 [74.88‒124.75]	0.368
Surgical procedure (n)				1.000
Hepatic cuneiform resection	0.0% (0)	0% (0)	0% (0)	1.000
Segmentectomy	73.9% (17)	73.9% (17)	73.9% (17)	
Hemihepatectomy	26.1% (6)	26.1% (6)	26.1% (6)	
Duration of surgery (min)	272 [203‒336]	249 [219‒391]	278 [225‒322]	0.302

Note: Data are presented as number (%) or means ± SD or median (1st quartile and 3rd quartile), as appropriate.

POD3, Postoperative Days-3; POD7, Postoperative Days-7; BMI, Body Mass Index; TBIL, Total Bilirubin; DBIL, Direct Bilirubin; TP, Total Protein; ALB, Albumin; ALT, Alanine Transaminase; AST, Aspartate Transaminase; GGT, Gamma-Glutamyl Transferase; ALP, Alkaline Phosphatase.

### *Effect of steatosis on postoperative IRI in patients underwent VIO*

Furthermore, we analyzed the effects of hepatic VIO on postoperative liver injury in different liver disease status ‒ steatosis or cirrhosis ‒ at POD3 or POD7 following liver resection with VIO, as reflected by ALT and AST^22^ (Fig. 2 and Table 3). Consistent with previous study, the status of cirrhosis had no significant effect on postoperative liver injury (data not shown).^15^ Considering steatosis, LIRI was significantly more severe in patients with steatotic liver, as evidenced by significantly elevated ALT levels on both POD3 (252.00 [115.60‒563.50], p = 0.026) and POD7 (118.00 [71.50‒187.80], p = 0.006), with a more pronounced trend observed on POD7. The number of occlusions did not differ significantly between the two groups. Among patients who underwent VIO, intraoperative blood loss was significantly greater in patients with hepatic steatosis compared with those without steatosis (583.33±340.68 mL vs. 358.33 ± 261.84 mL, p = 0.045), likely attributable to the more friable consistency of steatotic liver tissue. Notably, in our practice, transfusion was administered promptly when hemoglobin levels dropped below 70 g/L or when acute blood loss exceeded 20% of total blood volume, in accordance with institutional protocols.^23^ This strategy effectively prevented hypotension-induced exacerbation of liver injury that might otherwise result from excessive blood loss. Thus, IRI attributable to blood loss could be excluded, and the predominant causes of hepatic IRI were identified as hepatic steatosis and VIO.

**Fig. 2:**
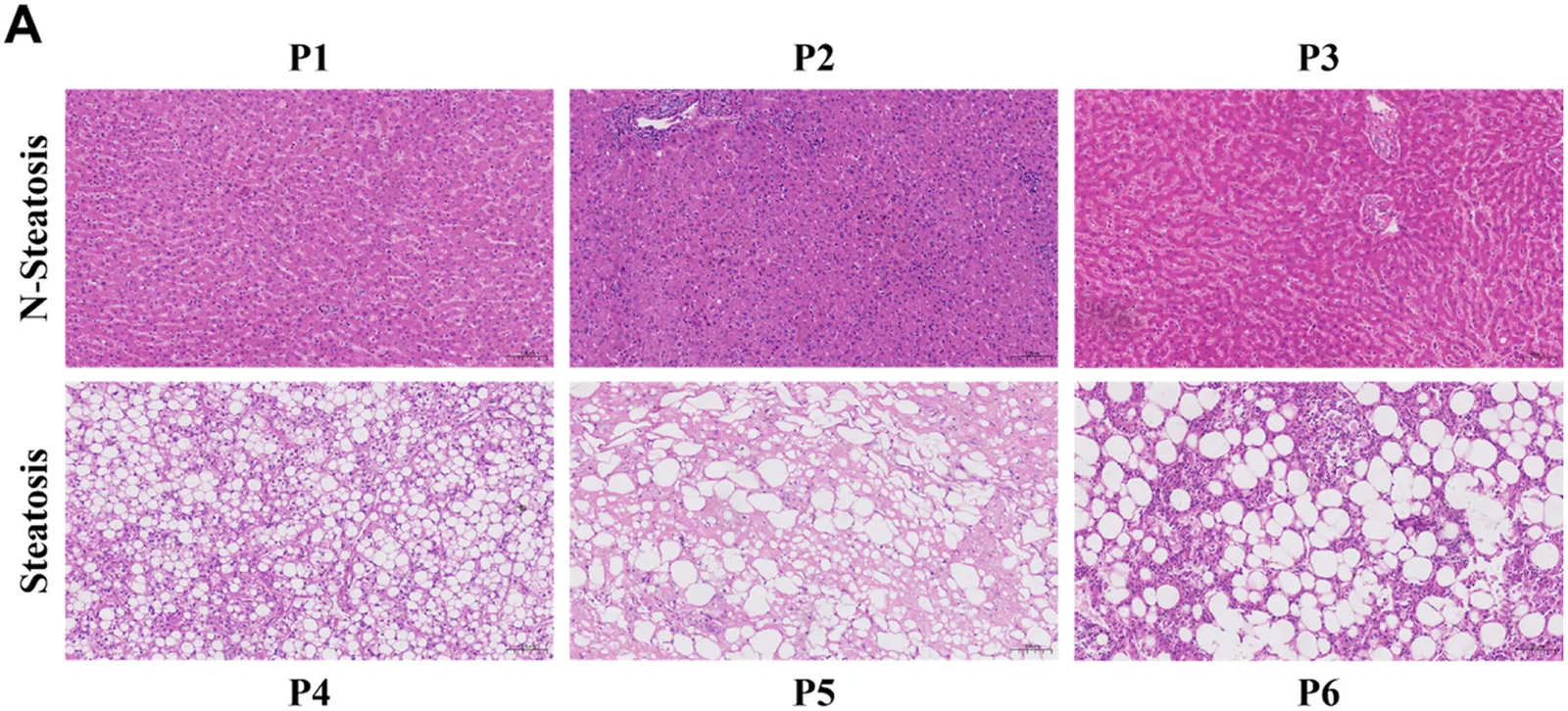
**Representative H&E-stained liver tissue sections from patients with and without hepatic steatosis.** Non-steatotic liver tissue (n = 3 representative cases) and steatotic liver tissue (n = 3 representative cases). Steatotic liver tissue demonstrates significant macrovesicular steatosis. The affected hepatocytes contain clear, rounded cytoplasmic vacuoles that displace the nuclei to the periphery, consistent with a diagnosis of > 5% hepatic fat accumulation. (P1‒6: Patient 1‒6; Scale bars: 100 μm).

**Table 3:** Comparison of liver function markers of different phases between n-steatotic and steatotic patients in the VIO group through PSM cohort study.

Indexes	N-Steatosis group (n = 12)	Steatosis group (n = 12)	p-value
Intraoperative blood loss (mL)	358.33±261.84	583.33±340.68	0.045*
Number of occlusions	2.0 [1.0‒2.5]	2.5 [1.5‒4.5]	0.304
Preoperative laboratory values			
ALT (U/L)	25.08±13.71	33.78±19.44	0.139
AST (U/L)	27.00 [19.53‒37.80]	27.90 [143.80‒541.50]	0.814
POD3 laboratory values			
ALT (U/L)	106.40 [57.13‒186.00]	252.00 [115.60‒563.50]	0.026*
AST (U/L)	86.50[34.50‒176.08]	185.00 [113.00‒286.00]	0.091
POD7 laboratory values			
ALT (U/L)	38.95[24.75‒51.90]	118.00 [71.50‒187.80]	0.006*
AST (U/L)	29.55 [24.35‒38.80]	50.00 [39.80‒75.40]	0.016*
Surgical procedure (n)			1.000
Hepatic cuneiform resection	25.0% (3)	25.0% (3)	
Segmentectomy	58.3% (7)	58.3% (7)	
Hemihepatectomy	16.7% (2)	16.7% (2)	

Note: Data are presented as number (%) or means ± SD or median (1st quartile and 3rd quartile), as appropriate.

POD3, Postoperative Days-3; POD7, Postoperative Days-7; ALT, Alanine Transaminase; AST, Aspartate Transaminase.

To exclude the effects of surgery and anesthesia, we compared the severity of liver damage between patients with hepatic steatosis and those without hepatic steatosis in the N-VIO group (Table 4). In this subgroup, intraoperative blood loss did not differ significantly between the two groups. Results showed no difference in ALT levels between the two groups on both POD3 (115.65 [48.00‒219.00] vs. 68.70 [39.20‒150.70], p = 0.773) and POD7(36.20 [29.85‒90.63] vs. 46.00 [29.00‒74.85], p = 0.910). These results indicate that hepatic steatosis is significantly able to increase liver sensitivity to IRI, placing patients with steatosis at greater risk of liver injury and dysfunction after hepatectomy involving VIO.

**Table 4:** Comparison of liver function markers of different phases between n-steatotic and steatotic patients in the N-VIO group through PSM cohort study.

**Indexes**	**N**‒**Steatosis group** **(n=28)**	**Steatosis group** **(n=28)**	**p‒value**
Intraoperative blood loss (mL)	100 [50‒275]	100 [22.5‒237.5]	0.951
Preoperative laboratory values			
ALT (U/L)	31.90 [15.68‒58.73]	35.25 [21.10‒60.08]	0.733
AST (U/L)	29.65 [20.15‒53.95]	30.20 [24.15‒40.60]	0.785
POD3 laboratory values			
ALT (U/L)	115.65 [48.00‒219.00]	68.70 [39.20‒150.70]	0.773
AST (U/L)	124.80 [50.90‒195.40]	67.70 [32.60‒168.90]	0.513
POD7 laboratory values			
ALT (U/L)	36.20 [29.85‒90.63]	46.00 [29.00‒74.85]	0.910
AST (U/L)	33.10 [26.40‒57.70]	24.70 [20.50‒58.15]	0.975
Surgical procedure (n)			1.000
Hepatic cuneiform resection	50.0% (14)	50.0% (14)	
Segmentectomy	14.3% (4)	14.3% (4)	
Hemihepatectomy	35.7% (10)	35.7% (10)	

Note: Data are presented as median (1st quartile and 3rd quartile), as appropriate.

POD3, Postoperative Days-3; POD7, Postoperative Days-7; ALT, Alanine Transaminase; AST, Aspartate Transaminase.

## Discussion

This study offered important insights into the clinical implications of VIO, particularly in the presence of steatosis. We found no significant difference in intraoperative blood loss and postoperative liver function among the N-VIO, F-VIO, and M-VIO groups. And the level of ALT is currently recognized as a diagnostic criterion for liver injury.^24^ Although the trend of higher median values in the VIO group persisted, statistically non-significant after PSM. The VIO does not cause additional liver damage during hepatectomy. It highlights the critical importance of the surgeon's technical strategy in minimizing procedure-related hepatic injury during hepatectomy. These results are consistent with previous studies indicating that the number of occlusion is not significantly associated with liver injury.^25^ However, some studies indicate that IRI is a prominent complication of hepatic resection when VIO is employed, so we further examined the relationship between the two based on disease stratification.^26^

Patients with pre-existing chronic liver disease, particularly those with hepatic steatosis, exhibit heightened susceptibility to Vascular Inflow Occlusion (VIO)-induced IRI during hepatectomy.^15^ Emerging evidence has firmly established fatty liver as an independent risk factor that amplifies the severity of IRI.^27^ Clinical studies have demonstrated that steatotic livers experience significantly exacerbated hepatocellular damage following VIO,^27^^,^^28^ which is also illustrated by the results of our clinical study. According to previous studies, the main mechanism usually involved in this process are macrophage polarization and inflammasome formation.^29^^,^^30^ Although the number of occlusions did not differ significantly between steatotic and non-steatotic livers, intraoperative blood loss was significantly greater in the steatotic group, likely attributable to the more friable consistency of steatotic liver tissue, which renders it more susceptible to bleeding during parenchymal transection. Notably, in the subgroup of patients who did not undergo VIO, blood loss was comparable between steatotic and non-steatotic livers, which may be explained by the relatively minimal blood loss in these patients, obviating the need for inflow occlusion and thus diminishing the observable impact of steatosis on bleeding. Therefore, this result should draw enough attention from patients with hepatic steatosis. Surgeons must recognize that while conventional clamping protocols are generally safe in healthy livers, they may exceed the ischemic tolerance of fatty liver tissue. Therefore, strict limitation of both cumulative occlusion time and the number of intermittent cycles should be implemented when VIO is deemed unavoidable in patients with fatty liver disease. Future research should focus on exploring adjunctive hepatoprotective strategies to mitigate IRI in this high-risk cohort.

Despite the above insights, the present work has both strengths and limitations. Its strengths lie in the consecutive, single-center cohort of 200 patients, the prospective-style capture of serial ALT/AST, and the explicit stratification by steatosis. Limitations remain, however. The retrospective design precludes causal inference, and long-term oncologic or functional outcomes were not examined.

## Conclusion

In sum, more pronounced liver injury is more likely attributable to differences in the surgical procedure rather than VIO. VIO does not exacerbate liver damage and IRI. Steatosis independently intensifies IRI, underscoring the need for stricter occlusion limits in steatosis. We therefore recommend that surgeons need not be concerned about VIO causing additional liver damage and should perform VIO when indicated.; when encountering patients with hepatic steatosis, greater caution should be exercised in administering VIO.

## Authors’ contributions

Hui Chen: Data curation; formal analysis; writing-original draft. Biao Duan: Data curation; formal analysis; writing-original draft. Shiyu Yang: Data curation; formal analysis. Nuo Pan: Data curation; formal analysis.Kun Tao: Investigation; validation. Xiaohui Liu: Investigation; validation. Bing Hu: Conceptualization; project administration; writing-review & editing. Jun Chen: Conceptualization; supervision; writing-review & editing. Weifeng Tan: Conceptualization; writing-review & editing; project administration; funding acquisition.

## Research ethics

This study was approved by the Ethics Committees of Shanghai Tongji General Hospital (October 19, 2024, n° K-2024–055). Written informed consent was obtained from all participants. This study was conducted in accordance with the Declaration of Helsinki (as revised in 2013). This study was conducted and reported in accordance with the STROBE (Strengthening the Reporting of Observational Studies in Epidemiology) guidelines.

## Informed consent

Informed consent was obtained from all individuals included in this study, or their legal guardians or wards.

## Declaration of generative AI and AI-assisted technologies in the writing process

During the preparation of this work, the authors did not use any generative Artificial Intelligence (AI) or AI-assisted technologies in the writing process, including but not limited to the drafting of the manuscript, data analysis, figure generation, or literature review. All content is the original work of the authors, and the authors take full responsibility for the integrity and accuracy of the content.

## Reporting guidelines

This study was conducted and reported in accordance with STROBE (Strengthening the Reporting of Observational Studies in Epidemiology) guidelines.

## Funding

This study was supported by National Natural Science Foundation of China (82,170,412) and the Tongji Hospital Affiliated to Tongji University funds for scientific research (RCQD2405).

## Conflicts of interest

The authors declare no conflicts of interest.

## Data Availability

Raw data were generated at Hepatobiliary Surgery Center, Tongji Hospital Affiliated to Tongji University, Shanghai. Derived data supporting the findings of this study are available from the corresponding author on request.
